# Qualitative system dynamics modelling to support the design and implementation of tuberculosis infection prevention and control measures in South African primary healthcare facilities

**DOI:** 10.1093/heapol/czae084

**Published:** 2024-08-31

**Authors:** Karin Diaconu, Aaron Karat, Fiammetta Bozzani, Nicky McCreesh, Jennifer Falconer, Anna Voce, Anna Vassall, Alison D Grant, Karina Kielmann

**Affiliations:** Institute for Global Health and Development, Queen Margaret University, Queen Margaret University Drive, Musselburgh EH21 6UU, United Kingdom; Institute for Global Health and Development, Queen Margaret University, Queen Margaret University Drive, Musselburgh EH21 6UU, United Kingdom; TB Centre, London School of Hygiene & Tropical Medicine, Keppel St, Bloomsbury, London WC1E 7HT, United Kingdom; TB Centre, London School of Hygiene & Tropical Medicine, Keppel St, Bloomsbury, London WC1E 7HT, United Kingdom; TB Centre, London School of Hygiene & Tropical Medicine, Keppel St, Bloomsbury, London WC1E 7HT, United Kingdom; Institute for Global Health and Development, Queen Margaret University, Queen Margaret University Drive, Musselburgh EH21 6UU, United Kingdom; School of Nursing and Public Health, College of Health Sciences, University of KwaZulu-Natal, 238 Mazisi Kunene Rd, Glenwood, Durban 4041, South Africa; TB Centre, London School of Hygiene & Tropical Medicine, Keppel St, Bloomsbury, London WC1E 7HT, United Kingdom; TB Centre, London School of Hygiene & Tropical Medicine, Keppel St, Bloomsbury, London WC1E 7HT, United Kingdom; Africa Health Research Institute, Nelson R. Mandela School of Medicine, University of KwaZulu-Natal, 719 Umbilo Road, Durban 4013, South Africa; Institute for Global Health and Development, Queen Margaret University, Queen Margaret University Drive, Musselburgh EH21 6UU, United Kingdom; Department of Public Health, Institute of Tropical Medicine, Nationalestraat 155, Antwerp 2000, Belgium

**Keywords:** System dynamics modelling, participatory group model-building, tuberculosis, infection prevention and control, South Africa, nosocomial transmission

## Abstract

Tuberculosis infection prevention and control (TB IPC) measures are a cornerstone of policy, but measures are diverse and variably implemented. Limited attention has been paid to the health system environment, which influences successful implementation of these measures. We used qualitative system dynamics and group-model-building methods to (1) develop a qualitative causal map of the interlinked drivers of *Mycobacterium tuberculosis* (*Mtb*) transmission in South African primary healthcare facilities, which in turn helped us to (2) identify plausible IPC interventions to reduce risk of transmission. Two 1-day participatory workshops were held in 2019 with policymakers and decision makers at national and provincial levels and patient advocates and health professionals at clinic and district levels. Causal loop diagrams were generated by participants and combined by investigators. The research team reviewed diagrams to identify the drivers of nosocomial transmission of *Mtb* in primary healthcare facilities. Interventions proposed by participants were mapped onto diagrams to identify anticipated mechanisms of action and effect. Three systemic drivers were identified: (1) *Mtb* nosocomial transmission is driven by bottlenecks in patient flow at given times; (2) IPC implementation and clinic processes are anchored within a staff ‘culture of nominal compliance’; and (3) limited systems learning at the policy level inhibits effective clinic management and IPC implementation. Interventions prioritized by workshop participants included infrastructural, organizational and behavioural strategies that target three areas: (1) improve air quality, (2) improve use of personal protective equipment and (3) reduce the number of individuals in the clinic. In addition to core mechanisms, participants elaborated specific additional enablers who would help sustain implementation. Qualitative system dynamics modelling methods allowed us to capture stakeholder views and potential solutions to address the problem of sub-optimal TB IPC implementation. The participatory elements of system dynamics modelling facilitated problem-solving and inclusion of multiple factors frequently neglected when considering implementation.

Key messages

- Implementing policies and measures to address tuberculosis (TB) transmission in health facilities is often done without consideration of local constraints.
- In South Africa, we collaborated with health workers, patients and other local stakeholders to design and contextualize interventions to reduce TB transmission in primary care clinics.
- Participatory group model-building and qualitative system dynamics modelling may be useful for developing complex, context-specific interventions to reduce TB transmission.

## Introduction

Tuberculosis (TB) is the second leading infectious cause of death after coronavirus disease (specifically the COVID-19 pandemic) and was responsible for 1.3 million deaths in 2022 (World Health Organization (WHO), 2023), the majority in low- and middle-income countries (LMICs). Transmission of *Mycobacterium tuberculosis* (*Mtb*) within health facilities is well-documented; of particular concern is transmission of drug-resistant *Mtb* (causing DR-TB) (Pearson *et al*., 1992). This is evidenced by the persistently high rates of TB infection and disease in health workers in high-TB-burden countries (Grobler *et al*., 2016; Uden *et al*., 2017; World Health Organization (WHO), 2020) and by outbreaks in health facilities (Gandhi *et al*., 2013).

Nosocomial transmission of *Mtb* is acknowledged to be driven by an interconnected set of factors, including limited ventilation, frequent and prolonged personal contact with infected individuals and poor adoption and adherence of personal hygiene measures (Fox *et al*., 2021). Infection prevention and control (IPC) measures are a cornerstone of policies intended to reduce *Mtb* transmission in healthcare and other ‘congregate’ settings (World Health Organization (WHO), 2020). However, while IPC measures are proven to reduce the *Mtb* burden associated with nosocomial transmission (Fox *et al*., 2021; Paleckyte *et al*., 2021), little is known about how such measures can be introduced in low-resource settings.

TB IPC consists of a package of diverse measures (World Health Organization (WHO), 2020), the implementation of which depends on the underlying capacities and dynamics of health systems (Claassens *et al*., 2013; O’Hara *et al*., 2017; Arakelyan *et al*., 2022). Zwama *et al*. (2021) highlight that TB IPC measures can be reframed as complex interventions, whose shape and implementation are dependent on diverse health system influences, including availability and functionality of existing infrastructure, existing culture of care, service delivery processes, as well as broader policy formulation. Most literature considers the extent to which national policies cover and include TB IPC measures, but far less emphasis is placed on the policy-to-implementation gap and monitoring and evaluation of such measures (Zwama *et al*., 2021).

As per the Consolidated Framework for Implementation Research (CFIR) (Damschroder *et al*., 2009), successful intervention implementation depends not only on the direct mechanisms of an intervention but also on the broader conditions for implementation and the ways in which these shape intervention mechanisms. Conditions refer to both internal and external settings surrounding the intervention and its implementation (i.e. the structural, political, cultural and organizational and wider policy, economic and social contexts), the values, motivations and goals of actors involved in carrying out the intervention or more broadly creating the space or timing conducive for implementation, as well as the active change processes required to cement the intervention as routine practice. Implementation science frameworks and approaches emphasize the need to consider how complex interventions interact with complex contexts and determinants (Damschroder *et al*., 2009; Northridge and Metcalf, 2016).

Northridge and Metcalf (2016) point to the utility of systems science approaches that consider systems with multiple components and the potentially non-linear and dynamic interactions between components. Accounting for such non-linear interactions is necessary particularly for complex interventions intended for implementation in complex health system contexts; previous research suggests that many interventions produce limited, counter-intuitive or incoherent results due to failure to accurately identify and account for complex influences on implementation (Braithwaite *et al*., 2018; Sheldrick *et al*., 2021).

The South African primary healthcare (PHC) facilities in which this study was undertaken represent complex contexts. PHC facilities in South Africa, particularly in rural areas, may provide not only acute care but also short- and medium-term specialist care (e.g. around pregnancy and tuberculosis) and long-term care for a large, growing population with chronic conditions such as human immunodeficiency virus (HIV), hypertension and diabetes mellitus (Mckenzie *et al*., 2017; Wong *et al*., 2021). Previous authors have discussed the complexity of working in these environments and have suggested that effective facility-level implementation requires consideration of the hierarchical and disease programme-focused structure of the health system and the resulting limited autonomy of ‘lower-level’ actors (Kawonga *et al*., 2016; Kielmann *et al*., 2021; Arakelyan *et al*., 2022) and also facility-specific characteristics such as organizational culture, quality and type of leadership (Lebina *et al*., 2020), service fragmentation, adequacy of physical and digital infrastructure (Malakoane *et al*., 2020), local geography and climate, knowledge and skill gaps, staff shortages and bureaucracy (Kredo *et al*., 2020).

System dynamics modelling (SDM) is a complexity science method increasingly applied in health policy and systems research (Chang *et al*., 2017; Cassidy *et al*., 2019; Darabi and Hosseinichimeh, 2020). Researchers note the method’s utility in identifying complex interactions between intervention and health system components and its potential for explicitly considering such interactions in intervention design and evaluation (Cassidy *et al*., 2022). Drawing on participatory group model-building (GMB) workshops with local stakeholders, the paper identifies the principal dynamic drivers of *Mtb* transmission in PHC facilities and discusses how they relate to health system and policy influences. The paper further details the process of using data derived from the GMB workshops to prioritize potential interventions to improve TB IPC at the primary care level.

## Methods

### Overarching study

This case study was embedded within a broader research project, ‘Umoya omuhle’ (Yates *et al*., 2023), which adopted a multidisciplinary, whole systems approach (Kielmann *et al*., 2020) to identify drivers of, and interventions suitable for addressing, *Mtb* transmission in PHC facilities in two provinces of South Africa [KwaZulu-Natal (KZN) and Western Cape (WC)].

### Design and aims

We adopted participatory GMB methods and followed the methods used in qualitative SDM (Rouwette *et al*., 2000; 2002; Sterman, 2000; Andersen *et al*., 2007). GMBs enable stakeholders’ embedded local contexts to explore and visually map dynamic relationships across inter-related and intersecting factors contributing to the emergence of specific challenges. This study aimed to identify the diverse contextual and infrastructural, environmental, behavioural, policy-related and community and health systems drivers of transmission of *Mtb* in South African PHC facilities. By identifying the underlying key dynamics that drive the emergence of the problem (transmission of *Mtb* in PHC facilities) and facilitating discussion around potential areas suited for intervention, the study further aimed to elicit context-appropriate intervention mechanisms, which could form the basis for further quantitative modelling (specifically mathematical and cost-effectiveness modelling). GMB was chosen for this project as it allowed a participatory and problem-focused approach to gathering data on nosocomial transmission in primary care; in contrast to traditional focus groups, the method places significant emphasis on participants co-creating products [such as causal loop diagrams (CLDs) reflecting qualitative insights gathered] that can depict the complex interactions of the broader systems that participants are embedded in. A key advantage of the method is producing outputs that can mobilize public health action (Gerritsen *et al*., 2020).

### Settings

Two settings with contrasting TB epidemiological profiles were chosen. KZN Province is one of four high-TB-burden provinces, with estimated drug-sensitive *Mtb* incidence of 525 per 100 000 population in 2017 (Day *et al*., 2019). The effective *Mtb* treatment coverage for KZN has been estimated at 56% for the 2016–18 triennium (Day *et al*., 2019). Initiation and management of drug-sensitive TB has been decentralized to the clinic level, while the initiation and continuation of multidrug-resistant TB (MDR-TB) has been decentralized to Level 1/District Hospitals (KwaZulu-Natal Department of Health, 2020).

WC province (population ∼6.6 million) is generally considered to have better health infrastructure than the rest of South Africa. Although it has fewer PHC clinics per capita than KZN (population ∼11.6 million; 1 clinic per 25 000 people in WC vs per 20 000 in KZN), many more of its clinics are community health centres (28% WC vs 4% KZN), which are usually larger and offer a wider range of services (Mckenzie *et al*., 2017). Six facilities per province were chosen for inclusion in ‘Umoya omuhle’ based on a range of criteria that included size, governing authority, location (urban vs rural), age and whether the clinic was currently treating individuals with DR-TB. Furthermore, we also considered whether facilities were implementing the Ideal Clinic initiative—recently introduced in South Africa to streamline health services at the primary care level and ensure more efficient service utilization (Matsoso *et al*., 2018)— and whether they had access to appointment systems and were part of the Centralized Chronic Medication Dispensing and Distribution (CCMDD) programme (Liu *et al*., 2021). Through either of these initiatives, we expected to see differences in the way patients were accessing services at the clinic.

### GMB workshops

We convened two 1-day GMB workshops in August 2019: one with policymakers and decision makers at national and province levels (Day 1, ‘policymakers’) and one with health professionals active at PHC facilities and district-level and patient advocates (Day 2, ‘practitioners’). Researchers from ‘Umoya omuhle’ also took part in each of the two workshops, feeding in research evidence from the broader project into debates.

We purposively targeted a diverse group of participants to capture a range of insights relevant to the complex problem of TB IPC implementation in the South African context. Overall, 9 policymakers and 15 practitioners took part in the workshops. All provided written informed consent. A full list of participant categories taking part in each workshop is presented in Table 1, and further details on participant sampling and selection are included in Supplementary File 2.

**Table 1.** Workshop aims, participants, scripts and activities

Workshop participants and aims	Participants	Scripts and activities used
Workshop 1: policy and decision-making stakeholders aim to identify broader policy influences relating to TB care systems and nosocomial transmission of TB, including potential interventions for addressing this.	Total: *n* = 9 Policymakers with expertise and roles across primary care, information systems, pharmaceutical management, service delivery and health financing (*n* = 9; details further omitted due to risk of identification).	Expectations of the day Reference modes: participants asked to draw graphs over last 10 years depicting (a) prevalence of DS and DR-TB, (b) overall ability of health system to respond, (c) policy interest in IPC and (d) policy interest in occupational health. Variable elicitation: participants use post-it notes to identify (a) drivers of TB, (b) factors affecting system’s ability to respond to patient needs and (c) policy drivers affecting TB and HIV care systems. Causal loop model: participants prompted to build a causal loop model, using elicited variables, and considering system hardware and software, in addition to wider policy, governance and financing issues. Points of fragility and intervention: participants each allocated 5 ‘points’ (blue stickers) to variables they felt corresponded to the most fragile areas in the system and 5 ‘points’ (red stickers) to the variables they felt corresponded to areas of potential intervention in the system. Elaboration of constraints: participants listed criteria used by stakeholders in prioritizing interventions and identified which of these were constraints on the system. Any further constraints were additionally listed. Intervention listing: participants brainstormed a list of interventions targeting points of fragility and areas of potential intervention. Policy and intervention prioritization: participants voted on the highest impact interventions and those interventions that would be most difficult to implement, with five green stickers and five red stickers, respectively, allocating points as they preferred.
Workshop 2: practice stakeholders, i.e. health professionals at facility and district levels, and patient advocates aim to depict dynamics of nosocomial transmission in clinics, including potential interventions for addressing this.	Total: *n* = 15 ‘KZN’: Province-level management, monitoring and leadership, representatives of architecture and infrastructure (*n* = 5) TB survivor and patient advocate (*n* = 1) Facility staff and leadership (*n* = 2) ‘WC’: Province-level management, monitoring and leadership (*n* = 1) TB survivor and patient advocate (*n* = 1) Facility staff and leadership (*n* = 2) ‘Not affiliated’: Architecture and infrastructure representatives (*n* = 2) TB survivor and patient advocate (*n* = 1) Additional: Umoya omuhle researchers (*n* = 2)	Rich pictures identifying transmission hot spots in clinics and reasons behind this. Graphs depicting participant impressions of trends of drug sensitive and drug resistant; how service provision evolved in relation to trends; wider interest in IPC over the last 20 years. Elicitation of variables relating to issues affecting TB transmission, factors affecting the TB patient pathway through clinics (from when patient arrives at clinic and then returns home/back to clinic for follow-up) and factors affecting a provider’s ability to respond to patient needs. Causal loop model elaboration to depict dynamics of system. Identification of weaknesses in overall system dynamics (points of fragility), areas where interventions may be suitable (areas of intervention) and criteria that constrain intervention impacts and feasibility. Elicitation of interventions targeting the areas identified above.

Abbreviations: DS, drug sensitive; RIMES, Research information, monitoring, evaluation and surveillance.

#### Workshop activities and analyses

Details of workshop aims, participants and activities are outlined briefly in Table 1 and described in detail in Supplementary File 2.

Analysis proceeded in an iterative manner. First, the research team collated materials produced in the workshops and transferred all to digital formats. The produced materials (graphs and rich pictures) were photographed, CLDs were transferred to Vensim and observer and reflector notes were imported to Microsoft Word. Second, a core modelling team (K.D., A.K., F.B. and J.F.) met to discuss the two CLDs produced in the workshops. The modelling team reviewed the phrasing of variables and pathways connecting these, considering the narratives captured in workshop notes. Where necessary, variable names and pathways were amended and diagrams were iteratively refined, with revisions clearly marked.

Third, follow-up calls were organized with workshop participants to secure feedback on these revisions, querying areas that remained unclear within diagrams and supplementing the latter with participant insights as relevant. Fourth, in line with research aims and system dynamics methodology, diagrams underwent stepwise reduction. The modelling team reviewed diagrams bearing in mind the boundary of the system to be modelled, i.e. the overarching scope of the problem of the risk of nosocomial transmission in PHC facilities and the potential for IPC-related interventions, and study aims to prospectively identify intervention mechanisms appropriate to context. Diagrams thus underwent a second round of simplification, whereby variables of distant importance to these challenges were deleted. Via a process of abduction involving repeated immersion in notes of the workshops and consideration of themes brought up by participants and triangulation of these against broader evidence globally and specific to South Africa, the modelling team further identified the probable core dynamics, i.e. pathways and feedback loops, contributing to both risk of nosocomial *MTb* transmission and implementation of IPC-related interventions. In online follow-up calls, participants and fellow researchers within the project were invited to offer feedback on these developments, with the core modelling team incorporating further insights as relevant.

In a fifth and final step, a broader research team (core modelling team and co-authors) then reviewed the intervention and fragility areas initially identified by participants, as well as the free-listed intervention mechanisms, and stepwise elaborated the steps by which these interventions would function and achieve their goals. In doing so, the researchers considered how the intervention mechanisms could build on the existing evidence base surrounding IPC interventions and their implementation within health systems (Zwama *et al*., 2021) and, where relevant, also reached out to further experts—e.g. on architecture, appointment and queuing systems. The pathways of action of the interventions were thus added to the identified feedback loops and intervention descriptions compiled; workshop participants and the wider ‘Umoya omuhle’ research group were invited to review and iteratively revise these, drawing as necessary on wider data available from the wider Umoya omuhle project. Elaboration of interventions was thus guided both by the context-specific evidence gathered by the project and GMB workshops and their outputs.

## Results

To offer insights into overarching context, we first outline descriptive findings relating to the exercises conducted prior to the elaboration of the CLDs. We then present these diagrams and describe the three main feedback loops identified as driving system behaviour. These diagrams correspond to the qualitative data obtained from the SDM GMB workshops described earlier and have not been quantitatively validated. Finally, we offer a summary of prioritized interventions in light of intervention mechanisms suggested by workshop participants.

### Policy and clinic context surrounding drivers of Mtb transmission and IPC implementation

**Figure 1.**
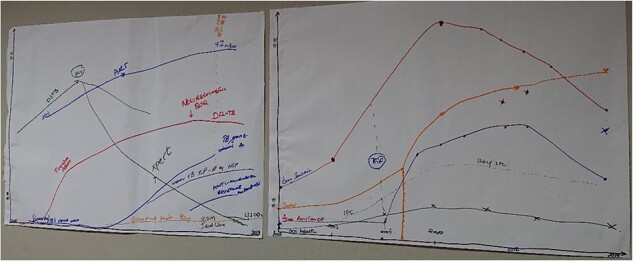
Graphs developed at GMB workshops

Policymakers noted that over the period 2009–19, South African health system actors had become more aware of the burden of TB (see labelled trend lines in Figure 1). Increased interest was brought about not only by the advancement and diffusion of new TB diagnostics (Xpert MTB/RIF in particular) but also by broader global awareness of the dangers of antimicrobial resistance. Policymakers acknowledged that a previous major TB outbreak [known as the Tugela Ferry outbreak (Cooke *et al*., 2011)] played an important role in alerting those involved in TB service delivery of the need to manage the DR-TB burden and the critical implications for the system if such outbreaks were not contained.

Policymakers reflected that interest in IPC and occupational health more widely was initially low but had spiked temporarily following the Tugela Ferry outbreak before decreasing again. Participants attributed the decrease in interest to the challenges of implementing IPC and occupational health in facilities, noting numerous barriers to successful implementation of such interventions. Comparing developments in TB relative to other service areas, policymakers expressed pride in how the health system handled the HIV burden in the country, including expansion of access to antiretroviral therapy. Compared with HIV, they noted that TB was often neglected.

Trends depicted by the practitioners during the second GMB workshop mirror the above. Practitioners noted, however, that decentralization of TB services, alongside the introduction of Xpert MTB/RIF, also played an important role in accurately quantifying the TB burden in the country. Furthermore, this group emphasized that IPC interest was very low at the clinic level: in the absence of major events, ‘people are now immune’ (participant quote) to messaging about IPC.

**Figure 2.**
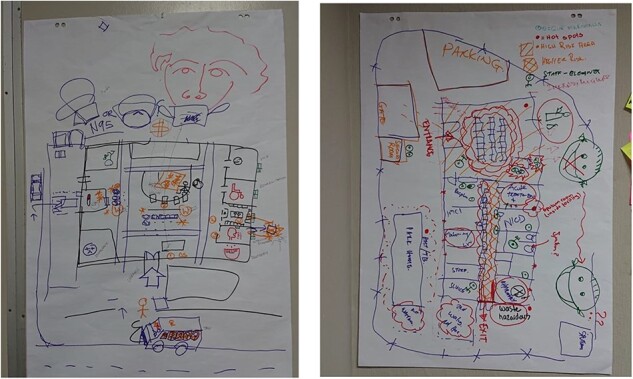
Rich pictures developed at GMB sessions depicting transmission hot spots in PHC facilities

Practitioners were also asked to elaborate rich pictures around what they considered to be transmission hot spots in facilities (Figure 2). Hot spots mentioned included waiting areas, medication and file pick-up spots, and areas where waste is collected and handled. Participants noted that healthcare workers (HCWs) and clerks are at high risk due to their prolonged exposure. Practitioners additionally stated that transmission is driven by a confluence of factors, including limited mask wearing at facilities (by both patients and HCWs) and infrastructure with limited ventilation (particularly in older buildings).

### Feedback loops influencing drivers of MTB transmission at the clinic level

Participants across GMB workshops offered an account of how drivers of *Mtb* transmission at the clinic level related to broader TB service delivery within PHC facilities and to policy influences. Later, we summarize the key dynamics and feedback loops identified following the analysis of these data.

#### Dynamic 1: MTB transmission is driven by bottlenecks in patient flow at given times

**Figure 3.**
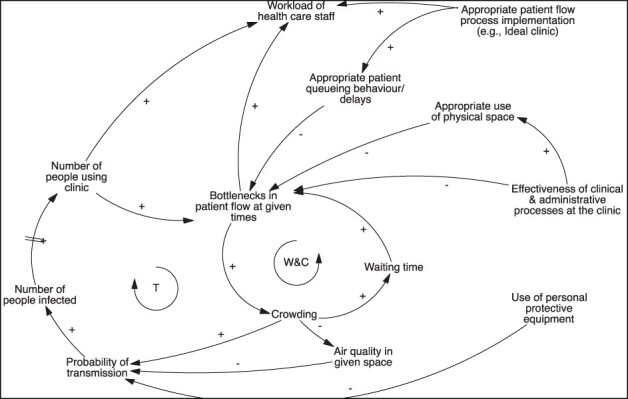
CLD—feedback loop surrounding probability of TB transmission in PHC facilities

Practitioners, in particular, noted that a key driver of *Mtb* transmission within facilities relates to the high number of people utilizing facilities at peak times and the service’s inability to see patients fast enough to prevent bottlenecks. Figure 3 shows two key feedback loops. The waiting time and crowding loop (W&C) is reinforcing. This loop highlights how, given high utilization of services at facilities in the early and mid-morning, bottlenecks in patient flow occur and crowds accumulate in specific clinic spaces. Overall, the waiting time increases for individuals joining the queue and, in the absence of mitigating action, contributes to bottlenecks persisting.

The ability of healthcare staff to address bottlenecks directly relates to the effectiveness of administrative and clinical processes. Administrative processes refer to the efficiency with which clerks can register patients, retrieve their files and direct them to appropriate spaces within the clinic, while clinical processes are directly dependent on the resources available at the clinic. For example, practitioners noted that staff and equipment shortages often mean that it is impossible to increase efficiency by seeing multiple patients simultaneously. Practitioners acknowledged that patient queuing behaviours also contributed to bottlenecks: patients face anxiety that they may not be seen and therefore prefer to queue close to the consultation room, thus causing crowding. Health practitioners and researchers noted, however, that if appropriate IPC processes were implemented, particularly patient flow processes such as the queuing and appointment systems envisioned by the Ideal Clinic initiative (Department of Health Republic of South Africa, 2022), bottlenecks may be prevented as patients could be more easily fast-tracked and triaged.

The second feedback loop we highlight is also reinforcing and relates to the increased risk of *Mtb* transmission within clinic spaces (T in Figure 3). As transmission increases, utilization of services in the long term also increases. This denotes an understanding by participants that if nothing was done to address current challenges, over time, it would be likely that more persons would acquire TB and more would also present at facilities requiring care. Participants believed the risk of transmission to be influenced by the number of people utilizing clinic services, the proximity between persons in clinic areas (e.g. waiting rooms) and the time spent in proximity. Air quality was noted to influence the risk of transmission, with health practitioners highlighting that few clinic spaces are appropriately ventilated (or that few facilities have outdoor spaces suitable for waiting) and that crowding itself reduces the quality of the air in a given environment. Use of personal protective equipment (surgical masks for patients and N95 respirators for health workers) was noted to be universally low, although acknowledged as one critical mitigation mechanism of *Mtb* transmission. These views were consistent with those expressed by HCWs and observed by ‘Umoya omuhle’ teams during ethnographic research in PHC facilities (Kallon *et al*., 2021).

#### Dynamic 2: implementation of IPC and effectiveness of clinic processes are anchored within a ‘culture of nominal compliance’

**Figure 4.**
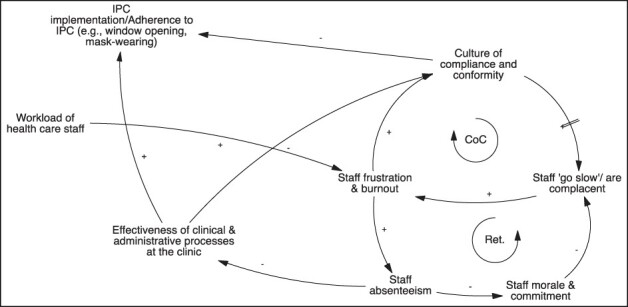
CLD—feedback loop surrounding a ‘culture of compliance’ in PHC facilities

Participants across the two GMB workshops offered accounts of the broader organizational climate surrounding TB service delivery and implementation of IPC at the primary care level. Figure 4 details two dynamics in this regard.

First, practitioners noted that high utilization of clinic services (including the emergence of bottlenecks in patient flows) directly influenced their workload and over time led to staff burn-out and frustration. The first reinforcing loop (‘Ret’ for retention in Figure 4) details how staff burn-out and frustration eventually lead to increased episodes of absenteeism, which in turn erode the morale and commitment of clinic-level staff in the long term. As morale decreases, staff are reported to become complacent and to ‘go slow’ (slow down and become unmotivated in their consultations), thus further creating resentment and tension and exacerbating the experience of burn-out among other staff at the clinic.

The second loop focuses on what participants termed a ‘culture of nominal compliance’ (CoC) that takes hold in facilities. Participants described how ‘going slow’ and burn-out are mutually reinforcing, prompting staff to eventually adopt a ‘ticking the boxes’ approach to IPC, rather than engaging in any reflexive practice. Workshop participants emphasized that ‘going through the motions’ of IPC implementation was compounded by an approach of mechanically conforming to guidelines and policy directives, rather than seeing IPC as a process for service improvement and for the protection of patients and staff.

Workshop participants expressed that corrective action by clinic leaders could help to balance the loops described earlier. If clinic managers were highly effective, identified problems early and intervened, the emergence of a CoC could be avoided. However, staff absenteeism itself compromises the effectiveness of clinic managers, e.g. by placing additional service provision demands on them. In situations where staff were absent or not performing, managers frequently reported to be ‘fire-fighting’. Reactive management, rather than more strategic and considered approaches, was therefore noted to be the norm.

#### Dynamic 3: limited systems learning inhibits effective clinic management and implementation of IPC

**Figure 5.**
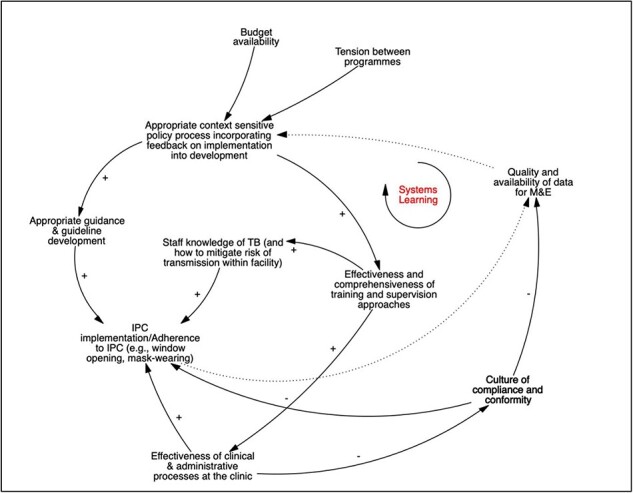
CLD—feedback loop surrounding the policy formulation process for TB IPC

Figure 5 offers an overview of distal macro-level influences on TB IPC implementation: specifically, the figure offers reflections on how the clinic and policy levels interact in influencing TB IPC policy and guideline development and implementation.

Policymakers reported an awareness of the CoC that had set in at facilities, particularly in relation to IPC. Participants from both policymaker and practitioner groups acknowledged that this was largely driven by the high demands placed on healthcare professionals at the clinic, but additionally noted that repeated reforms in the health system also likely contributed to staff burn-out. Particularly, the introduction of the Ideal Clinic initiative, which promoted a transition to integrated care and away from specialized and vertical service delivery, was noted to be challenging for healthcare staff.

Within this context, policymakers noted that the CoC at the clinic level extended to the quality and availability of data to inform policy formulation. Dotted lines in Figure 5 illustrate the information flows that ought to occur to inform policy response, whereas the systems learning feedback loop (noted as a loop) highlights challenges in relation to this. Workshop participants indicated moderate levels of mistrust in available data. Not only in the absence of high-quality and comprehensive data but also given limited participation of community groups and health staff in the policy formulation process, policymakers felt that their only choice was to design policy and guidelines building on best available international evidence. The group noted, however, that this meant that policy was often not informed by the realities of local implementation and that policy may be inappropriately formulated and not fit for context. Given limited feedback mechanisms and system learning, the policy process therefore essentially takes place without appropriately incorporating implementation insights.

Policymakers acknowledged the existence of the policy-implementation gap, noting that the current policy formulation process leaves little space for active change management. The latter would be a process whereby insights into the different aspects of any proposed policy change are considered alongside implementation challenges and revised and refined accordingly. One example of absent change management was noted by the research team during the workshops. Specifically, policymakers spoke of how IPC training and guidelines were introduced in the past. They noted that past policy cycles placed emphasis on the design and rollout of IPC guidelines and strengthening of staff knowledge around *Mtb* transmission. Practitioners, however, emphasized the need for problem-based training, on how to implement, monitor and supervise IPC implementation given the inadequate infrastructure and staff resistance to continued use of personal protective equipment [see the centre of the diagram (Figure 5)].

### Interventions for strengthening the TB IPC system in South African primary care facilities

**Figure 6.**
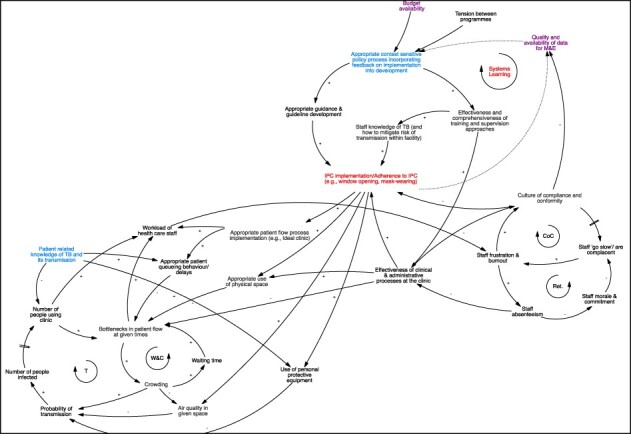
CLD (merged previous diagrams)

Figure 6 offers an overview of the dynamics described across previous sections and, as per workshop and follow-up call discussions, additionally highlights areas identified as particularly fragile within the existing system, areas requiring intervention and areas both fragile and of priority for intervention. See agenda for listing of variables.

Fragile areas are the systems learning loop, and the IPC implementation/adherence to IPC variable. Areas requiring intervention are the variables: Patient related knowledge of TB in transmission and Appropriate context sensitive policy process incorporating feedback on implementation into development. Variables that are both fragile but requiring intervention: Quality and availability of M&E and Budget availability.

In line with previous accounts, the figure draws attention to the patchy implementation of TB IPC in South African PHC facilities (see the centre diagram) and further highlights the multiple areas in need of immediate strengthening and intervention (in purple), including management of patient flow processes, CoC and effectiveness and responsiveness of training approaches. An area of intervention identified relates to patients’ knowledge of TB: across both GMB workshops, this was linked not only to patient health-seeking behaviour but also to their own personal adherence to IPC measures, including appropriate queuing and donning of surgical masks within facilities for both patient and health worker protection.

When identifying the points later, it is worth noting that despite some areas being priorities for intervention and intervention mechanism development, not all these were taken forward by participants. For example, participants reflected that strengthening the current policy-implementation gap would be a lengthy process and not feasible in the short term.

Table 2 offers an overview of the priority interventions proposed by workshop participants and elaborated in partnership with the ‘Umoya omuhle’ research team to reduce the risk of nosocomial *Mtb* transmission in South African PHC facilities. Initially, the intervention mechanisms were elaborated at the workshops, considering the dynamics discussed earlier and feasibility concerns. The modelling team refined intervention mechanisms leveraging insights from the broader ‘Umoya omuhle’ project and global evidence, as well as experts where needed. When elaborating interventions, the team considered higher-order interactions between the various feedback loops identified in the CLD; mechanisms that directly seek to counteract potential negative drivers are summarized under Unique and Shared Intervention Elements in Table 2. For example, to counteract a ‘CoC’, interventions include workshops where facility staff themselves can adapt and troubleshoot interventions, as well as further training and supportive monitoring and supervision. Further details of the interventions and modelling undertaken to estimate their likely impact on nosocomial Mtb transmission and their relative cost-effectiveness are the subject of other publications by our research team (McCreesh *et al*., 2021; Bozzani *et al*., 2023).

**Table 2.** Interventions elaborated by GMB participants and Umoya omuhle researchers (in brief)

Intervention title	1: Opening doors and windows	2: Building retrofits (e.g. raised roofs and lattice windows)	3: Installation of UVGI lights	4: Universal surgical mask wearing for patients and N95 respirators wearing for staff	5: Medication pick-up outside facilities	6: Queue management system	7: Appointment system
Target of intervention	Ventilation	Ventilation	Safety of spaces	Personal protection	Number of persons utilizing care at facility at given time	Number of persons queuing together at given time	Number of persons utilizing care at facility at given time
Type of intervention	Behaviour change	Infrastructure redesign	Infrastructure redesign	Behaviour change	Process and system change	Process and system change	Process and system change
Focus of the intervention and target variable in CLD	Changing ventilation by influencing IPC implementation and air quality in given space	Changing ventilation by influencing air quality in given space	Changing transmission risk and probability by influencing the safety of spaces	Changing probability of transmission by influencing the use of protective equipment	Changing the number of people utilizing services at the clinic at a given time and crowding by redirecting them to other spaces	Changing crowding in clinic spaces by promoting better queuing among patients, including outside clinics (outdoors)	Changing the number of people utilizing services at the clinic at a given time and crowding by regulating when patients present
Unique implementation steps	Weekly staff and monthly community workshops in first 3 months to enable troubleshooting + intervention being embedded	One-off workshop to decide which retrofits	UVGI installation + new operational manager responsibilities	Weekly staff and monthly community workshops in first 3 months to enable troubleshooting + intervention being embedded	Increase number of pick-up points under the CCMDD program; revise guidelines to increase the number of ART patients potentially eligible; increase medicine dispensation time frame	Staff manage queues at entry to facility, where possible outdoor waiting is put in place and patients are encouraged to wait there; queues are routinely monitored with patients reassured of place in queue	Different appointment slots put in place for different patient groups, with emphasis on patient communication and also punishment mechanisms for persons presenting outside slots (e.g. longer wait unless emergency)
Shared implementation steps	Training by the Office of Health Standards Compliance, at the facility level for all staff Training for IC peer reviewers and operational managers at the central level Supervision and monitoring by operational managers (with IPC lead support) Additional monitoring and communication to facility and policy via IC peer and HAST reviews Communication campaigns with patients and staff		Training by the Office of Health Standards	Training by the Office of Health Standards Compliance, at the facility level for all staff Training for IC peer reviewers and operational managers at the central level Supervision and monitoring by operational managers (with IPC lead support)		Workshops between facility staff and communities to decide on the design of the new systems/regularly check-in Training by the Office of Health Standards Compliance, at the facility level for all staff on necessity of new systems Training for IC peer reviewers, operational managers at the central level Supervision and monitoring by operational managers	
	Surveys with patient to monitor satisfaction and potential issues						

Abbreviations: ART, antiretroviral therapy; IC, ideal clinic; HAST, HIV/ Acquired immunodeficiency syndrome (AIDS)/STI/TB; STI, sexually transmitted infection; UVGI, ultraviolet germicidal irradiation.

## Discussion

Beckett *et al*. (2018) emphasizes how the research community needs to increasingly focus on the ‘messy reality of real-world health care’ in order to generate ‘rich, implementable knowledge for health care policy and practice’ (Beckett *et al*., 2018). Complexity science approaches are of potential benefit for implementation science (Northridge and Metcalf, 2016); however, to date, studies reflecting on the use of such methods for intervention design and development, or for studying policy implementation, are limited (Chang *et al*., 2017; Cassidy *et al*., 2019; Darabi and Hosseinichimeh, 2020). Our study provides an example of how SDM can be used to tackle messy problems and illustrates how participatory GMB was able to channel insights of diverse groups of stakeholders for identifying potential interventions able to tackle the problem of nosocomial TB transmission in a low-resource setting.

We identified three overarching dynamics influencing the risk of *Mtb* transmission at the clinic level. We further identified the broader organizational and policy context that drives *Mtb* transmission risk. Specifically, we identify how high utilization of clinic services at peak times often overwhelms existing capacity to address patient needs, resulting in long waiting times and bottlenecks. As waiting and crowding occur in areas that are poorly ventilated and relatively small, risk of *Mtb* transmission increases. We note that the capacity to address high patient utilization and implement IPC measures is critically dependent on not only levels of staffing but also motivation. Our findings identify that in some facilities, a CoC and conformity have set in, whereby implementation of IPC is one of many requirements made of staff who are facing burn-out due to high workload and implementation of new initiatives. Ethnographic field work conducted in these facilities corroborates these findings (Zinatsa *et al*., 2018; Kallon *et al*., 2021; Arakelyan *et al*., 2022), and broader global literature has also noted similar dynamics (Tan *et al*., 2020; Islam *et al*., 2021). At the macro level, we note that policymakers are aware of the difficulties in implementing IPC measures at the clinic level. However, given resource constraints and limited mechanisms for participatory policy development and learning from existing practices, policy formulation often does not bear implementation challenges in mind. Complementary studies with policymakers underline that there is no sense of urgency associated with policy formulation around IPC measures and that substantial barriers to mobilization around such policies exist (Colvin *et al*., 2021). Studies in other contexts have also identified similar challenges (Birgand *et al*., 2015).

Given these existing dynamics, which largely describe health system–related constraints on implementation of IPC [or the inner and outer settings of intervention implementation as per the CFIR (10)], we identified a suite of possible interventions that could (1) mitigate constraints and (2) promote active change processes to embed interventions over time so that they become routinely implemented.

Existing frameworks for implementation science, such as the CFIR (Damschroder *et al*., 2009), emphasize the need to carefully consider and develop intervention mechanisms and implementation steps, including active change management processes, that are suited and responsive to the organizational and broader social, policy and economic contexts within which they are implemented. The CFIR also emphasizes the need to focus on establishing the credibility of, and trust in, any intervention mechanisms proposed from the perspective of individuals involved in implementation or delivery (Damschroder *et al*., 2009). To this end, participatory SDM is particularly promising as it allows for the co-creation of interventions with those who will be ultimately involved in change processes, intervention delivery or potential scaling.

In this regard, qualitative SDM and participatory GMB—as used in this project—allowed us to dive into the problem of drivers of *Mtb* transmission in primary care and to capture multiple views on what is needed to address this problem. In contrast to other approaches, such as individual interviews or ethnographic research, the participatory elements of SDM facilitated a problem-solving approach and allowed for consideration of intangible factors that impact intervention success—both elements of potential benefit to implementation science (Northridge and Metcalf, 2016). For example, we identify the need for a culture of learning within the health system, whereby implementation-related insights can be mobilized to reframe guidelines and policy to become more context appropriate. As Northridge and Metcalf emphasize, such broad, non-quantifiable influences on implementation are often neglected when considering intervention development (Northridge and Metcalf, 2016).

By facilitating discussions regarding intervention approaches around a model that acts as a boundary object, SDM also allowed for diverse stakeholders to debate the shape of intervention mechanisms and promoted possibility thinking (Northridge and Metcalf, 2016). As such, SDM may be a useful tool for integrated knowledge translation (Beckett *et al*., 2018). An ancillary study to the one presented here actively considered the utility of SDM for both evidence generation and evidence translation in health policy (Perera *et al*., 2022). Based on findings of this study, we subsequently used mathematical and economic modelling techniques to simulate the impact of the interventions on *Mtb* transmission in facilities and community-wide TB incidence; we also estimated costs of interventions that included consideration of the enablers required to maintain some interventions and to estimate intervention cost-effectiveness (McCreesh *et al*., 2021; Bozzani *et al*., 2022).

In addition to notes included in the reflexivity statement, we acknowledge several limitations regarding this study. Participants had different ways in which they conceptualized both feasibility and potential intervention impact; both these considerations helped us narrow down which intervention mechanisms to further develop and to model quantitatively (Bozzani *et al*., 2023; McCreesh *et al*., 2021; McCreesh *et al*., 2022). Such discrepancies are more widely acknowledged in the literature (Vassall *et al*., 2019). However, in future research, it may be helpful to spend more focused time on aligning such considerations. Third, while the participatory workshops conducted in South Africa were well-attended, the follow-up check-in calls were done remotely and attended by fewer participants. Although we have fed back and validated findings with our participant group at subsequent research uptake events, it is still possible that the views expressed by the smaller group in the check-in calls unduly influenced intervention design. We also acknowledge that our GMB participants focused on managerial, programme and policymakers, thus potentially under-representing the views of front-line health workers and patients frequenting TB clinics. However, the Umoya omuhle project collected comprehensive qualitative data from clinic ethnographies and interviews with both health workers and patients. These data and findings derived from it were considered throughout the process of intervention elaboration. No formal community consultations took place. Fourth, while we made every attempt to include insights from global evidence into our qualitative models (e.g. ensuring that the intervention mechanisms were elaborated in line with global evidence), our models are still highly contextualized: our participants actively debated and challenged global evidence and adapted interventions according to their lived experience. Finally, it is important to acknowledge that we engaged only in qualitative SDM modelling and that the findings of our work reflect the contexts of the participants who participated in workshops. While our findings may not be generalizable, we consider that our approach, lessons documented here, and interventions elaborated may prove to be transferable to other settings.

## Conclusion

Using qualitative and participatory SDM allowed us to capture a range of perspectives on the problem of sub-optimal implementation of TB IPC measures in facilities, as well as potential interventions that could be implemented within the complex context of South African PHC facilities. The participatory elements of GMB facilitated a grounded problem-solving focus and allowed for consideration of often neglected factors that frequently affect implementation. In this project, SDM helped us to identify plausible interventions involving infrastructural, organizational and/or behavioural changes in health systems to support improved TB IPC. Our experience suggests that SDM can be a useful tool for stakeholder engagement in designing IPC interventions that take contextual complexity into account.

## Supplementary Material

czae084_Supp

## Data Availability

The data sets generated and analysed during this study will be made publicly available on the LSHTM repository: https://datacompass.lshtm.ac.uk.

## Reflexivity statement

This work was carried out as part of a multidisciplinary research partnership known as Umoya omuhle. A full list of partnership contributors is available in Supplementary File 1. We acknowledge that this publication specifically is reflective of the sample of participants involved in workshops as well as the orientation and positionality of the research and modelling team undertaking the work. While we attempted to capture all perspectives relevant to the problem under study and ensured that the modelling team was multidisciplinary, our own perspectives may have unduly influenced the research process and outputs. The authors include eight females and one male and span multiple levels of seniority. Only two authors had not previously conducted research in South Africa; all other authors had extensive experience conducting research in the setting on varied topics. Only two of the researchers had expertise in system dynamics; however, across the authorship group, diverse types of expertise were present, including qualitative and ethnographic research (four members), epidemiological research (three members), economic (two members) and mathematical modelling (three members) and implementation research (four members).
