# Supplementary material for: Qualitative system dynamics modelling to support the design and implementation of tuberculosis infection prevention and control measures in South African primary healthcare facilities
Source: Health Policy Plan. 2024 Aug 31;39(10):1041–54. doi: 10.1093/heapol/czae084 (PMC11562122; doi:10.1093/heapol/czae084)
Supplement: czae084_Supp [file czae084_supp.zip › Supplementary File 1.docx]

The extended *Umoya omuhle* team, institutions, and roles (listed alphabetically by surname)

| Name | Institution/s | Role |
| --- | --- | --- |
| Siphokazi Adonisi | UCT | Research Assistant |
| Kathy Baisley | LSHTM; AHRI | Co-investigator |
| Peter Beckwith | LSHTM; UCT | Research fellow |
| Fiammetta Bozzani | LSHTM | Co-investigator |
| Amy Burdzik | UCT | Occupational health |
| Adrienne Burrough | LSHTM | Project Manager |
| Nkosingiphile Buthelezi | AHRI | Research Assistant |
| Xolile Buthelezi | AHRI | Diagnostic Lab Manager |
| Ruvimbo Chigwanda | UCT | Administration |
| Christopher Colvin | UCT | Co-investigator |
| PIP CRAs | AHRI | Clinic research Assistants |
| Njabulo Dayi | AHRI | Research Data Manager |
| Arminder Deol | LSHTM | Mathematical modeller |
| Karin Diaconu | QMU | Co-investigator |
| Siphephelo Dlamini | AHRI | Nursing Manager |
| Yutu Dlamini | AHRI | Research Assistant |
| Raveshni Durgiah | AHRI | Grants office |
| Anita Edwards | AHRI | Head: Scientific Support |
| Jennifer Falconer | QMU | Research Assistant |
| Kitty Flynn | QMU | Administrator |
| Patrick Gabela | AHRI | Clinical Research Data Coordinator |
| Dickman Gareta | AHRI | Head: Research Data Management |
| Awethu Gawulekapa | UCT | Research Assistant |
| Harriet Gliddon | AHRI; UCL | Research Assistant |
| Bavashni Govender | UKZN | Administration |
| Indira Govender | LSHTM; AHRI | Co-investigator |
| Alison Grant | LSHTM; AHRI | Principal investigator |
| Meghann Gregg | LSE | Research Fellow |
| Emmerencia Gumede | AHRI | Research Assistant |
| Sashin Harilall | AHRI | Grants office |
| Kobus Herbst | AHRI | Chief Information Officer |
| Tamia Jansen | UCT | Research Assistant |
| Seonaid Kabiah | UCT | Research Assistant |
| Idriss Kallon | UCT | Post-doctoral researcher |
| Aaron Karat | LSHTM | Co-investigator |
| Hannah Keal | AHRI | Communications |
| Suzanne Key | UCT | Occupational health |
| Zama Khanyile | UKZN | Research Assistant |
| Mandla Khoza | AHRI | Clinic Research Assistant |
| Nozi Khumalo | AHRI | Systems Engineer |
| Zilethile Khumalo | AHRI | Research Assistant |
| Karina Kielmann | QMU | Co-principal investigator |
| Nondumiso Kumalo | AHRI | Clinic Research Assistant |
| Richard Lessells | AHRI | Epidemiologist |
| Nokuthula Lushaba (deceased) | UKZN | Administration |
| Sithembiso Luthuli | AHRI | Research Assistant |
| Sinethemba Mabuyakhulu | AHRI | Clinic Research Assistant |
| Hayley MacGregor | IDS | Co-investigator |
| Nonhlanhla Madlopha | AHRI | Research Assistant |
| Aphiwe Makalima | UCT | Administration |
| Tacha Malaza | AHRI | PIP CRA |
| Sifundesihle Malembe | AHRI | Research Assistant |
| Godfrey Manuel | UCT | Transport |
| Nonhlanhla Maphumulo | UKZN | Administration |
| Precious Mathenjwa | UCT | Research Assistant |
| Sanele Mbuyazi | AHRI | PIP CRA |
| Nicky McCreesh | LSHTM | Co-investigator |
| Claire McLellan | QMU | Administrator |
| Simphiwe Mdluli | AHRI | PIP CRA |
| Thabile Mkhize | AHRI | Transport |
| Duduzile Mkhwanazi | AHRI | Research Assistant |
| Zinhle Mkhwanazi | AHRI | Research Assistant |
| Zodwa Mkhwanazi | AHRI | Research Assistant |
| Anathi Mngxekeza | UCT | Research Assistant |
| Tshwaraganang Modise | AHRI | Research Data |
| Sashen Moodley | AHRI | Microbiology Laboratory Supervisor |
| Samantha Moyo | UCT | Research Assistant |
| Silindile Mthembu | AHRI | Clinic Research Assistant |
| Nozipho Mthethwa | AHRI | Research Assistant |
| Siphesihle Mthethwa | AHRI | Procurement Coordinator |
| Sphiwe Mthethwa | AHRI | Research Assistant |
| Sanele Mthiyane | AHRI | Research Assistant |
| Vanisha Munsamy | AHRI | Grants office |
| Sinead Murphy | UCT | Research Assistant |
| Thomas Murray | AHRI | Research assistant |
| Senzile Myeni | AHRI | PIP CRA |
| Tevania Naidoo | AHRI | Procurement |
| Nompilo Ndlela | AHRI | Research Assistant |
| Zama Ndlela | AHRI | PIP CRA |
| Thandekile Nene | AHRI | Research Assistant |
| Phumla Ngcobo | AHRI | Communications |
| Nzuzo Ntombela | AHRI | Research Data Systems Service Manager |
| Sabelo Ntuli | AHRI | GIS Coordinator |
| Nompumulelo Nyawo | AHRI | Human resources |
| Phumzile Nywagi | UCT | Research Assistant |
| Stephen Olivier | AHRI | Statistician |
| Justin Parkhurst | LSE | Co-investigator |
| Alex Pym | AHRI | Co-investigator |
| Yolanda Qeja | UCT | Research Assistant |
| Anand Ramnanan (deceased) | AHRI | Procurement |
| Sharmila Rugbeer | UKZN | Administration |
| Janet Seeley | LSHTM | Co-investigator |
| Aruna Sevakram | AHRI | Scientific support |
| Sizwe Sikhakane | AHRI | Transport |
| Zizile Sikhosana | AHRI | Somkhele Laboratory Supervisor |
| Theresa Smit | AHRI | Head: Diagnostic Research |
| Thandeka Smith | UKZN | Research Assistant |
| Naomi Stewart | LSHTM | Communications |
| Alison Swartz | UCT | Co-investigator |
| Amy Thomas | LSHTM | Communications |
| Siphosethu Titise | UCT | Research Assistant |
| Anna Vassall | LSHTM | Co-investigator |
| Marlise Venter | AHRI | Facilities Administrator |
| Anna Voce | UKZN | Co-investigator |
| Richard White | LSHTM | Co-investigator |
| Tom Yates | Imperial | Co-investigator |
| Precious Zulu | AHRI | Administration |
| Gimenne Zwama | QMU | Research Fellow |

AHRI: Africa Health Research Institute; IDS: Institute of Development Studies; LSE: London School of Economics and Political Science; LSHTM: London School of Hygiene & Tropical Medicine; QMU: Queen Margaret University; UCT: University of Cape Town; UKZN: University of KwaZulu-Natal
