# Supplementary material for: Qualitative system dynamics modelling to support the design and implementation of tuberculosis infection prevention and control measures in South African primary healthcare facilities
Source: Health Policy Plan. 2024 Aug 31;39(10):1041–54. doi: 10.1093/heapol/czae084 (PMC11562122; doi:10.1093/heapol/czae084)
Supplement: czae084_Supp [file czae084_supp.zip › Supplementary File 2_CLEAN.docx]

**Supplementary File 2: Further methodological details**

***Sampling and participants***

A first list of targeted participants was compiled by the research group. We were interested in capturing relevant policy and practice insights on the organisation of TB care. Participants included persons responsible for designing clinics and their infrastructure (e.g. architects working with the Ministry of Health) and health professionals working on the design of health services relating to TB and HIV and their integration into primary care (e.g. policymakers, representatives of key health system departments ranging from financing, to medicine and procurement, clinical care). To capture perspectives of clinic-based health professionals we invited nurses with TB-related responsibilities, and clinic as well as district health managers; occupational health representatives were also invited given the increased risks professionals involved in TB care face. Additionally, we invited patient advocates to capture health service user perspectives. Convenience sampling was further employed in instances where members of this first list could not attend the workshops. Participants were informed of the purpose of the study and research activities.

### Facilitation and modelling team

Following group model building practice, specific roles were assigned to research group members given available expertise. All team members received a briefing on their roles and responsibilities prior to the workshops and were invited to post-workshop debrief sessions to discuss facilitation and reflections on workshop content. [BLINDED] acted as modeller and facilitator, having had previous experience of both group model building and system dynamics studies. [BLINDED – 2 members] acted as facilitators and reflectors, KK as process coach and debriefer, [BLINDED] as recorder, and [BLINDED – 2 members] as gatekeepers given longstanding research engagement and work in South Africa. Two observers were additionally present at all sessions.

***Workshop activities***

Each workshop was structured around a series of activities bespoke to the targeted participant group and workshop aims. The workshop with policymakers focused on identifying the macro-level health system and policy influences on TB prevention and care (Vassall et al., 2016). Activities focused on identifying stakeholders’ perceptions of how the TB burden has evolved over time, and the concomitant capacity of the health system to address this burden and implement IPC and other occupational health measures. Further, via an exercise to elicit variables, participants were invited to reflect on key events and factors influencing the burden of TB in South Africa, including those related to existing TB and HIV care systems. The latter was chosen as a point of focus given the high number of people with TB and HIV in the population and policy emphasis on HIV in the local context.

Drawing on previously identified variables, an experienced SDM facilitator and modeller (KD) guided participants in the development of an initial causal loop diagram depicting the broader policy influences surrounding TB care systems, specifically focused on MTb transmission and its driver. Using this diagram, participants then identified priority areas of the depicted system which were weak (points of fragility) or potentially suitable for intervention (points of intervention). Participants then deliberated on criteria relevant for assessing the likely impact of interventions and feasibility of implementing these in the South African health system. They then were asked to free-list broad intervention mechanisms relevant to the prioritized fragility and intervention points. Participants then prioritised interventions they had identified in terms of perceived likely impact and feasibility of implementation, thus constantly reflecting on intervention implementation requirements and likely impacts during the workshops.

The workshop with practitioners included health professionals active at clinic and district levels. Workshop activities were similar to those outlined above, this time with a focus on identifying proximal factors related to TB care delivery and nosocomial transmission. The aim was to link these to distal elements reflective of both the South African context and health system, including available policies and guidance on IPC. The researcher-participants from Umoya omuhle and the facilitators were familiarised with the latter issues during the workshop in Day 1, and could thus prompt and challenge ongoing discussions to gain a broader understanding of dynamics played out at facility level. Similar to policymakers, practitioners were asked to use the proximal and distal variables they had identified and develop a causal loop diagram. Given the size of the group, three facilitators assisted in the building of the causal loop diagram and subsequent activities ([BLINDED – 3 members]), including, as in the previous day, an identification of points of fragility and intervention within the developed causal loop diagram and further a free listing and prioritization of interventions identified as suitable for addressing nosocomial transmission. At the outset of this workshop, participants were asked whether they wished to focus on province specific dynamics, or whether they wished to discuss and build models reflective of the reality of South African primary care facilities more generally: participants opted for the latter.

***Criteria for prioritisation of interventions***

During group model building workshops, participants were asked to free-list criteria that would aid them in prioritising interventions. Participants immediately identified criteria relevant to impact (e.g. reduced burden of disease, quality of care and patient satisfaction) as well as feasibility of implementing interventions (e.g. cost of introducing measures, challenges related to infrastructure or organisation of care, etc). Bearing likely impact and feasibility of implementation in mind, participants further prioritised the interventions. This additionally involved considering trade-offs between the different interventions and considered diverse types of impact.
